# Rapid Gel-Based Recombinase Polymerase Amplification Assay for Detection and Sequence Confirmation of *Ehrlichia canis*

**DOI:** 10.3390/ani16152443

**Published:** 2026-08-06

**Authors:** Peeravit Sumpavong, Kitjawan Khumtub, Krittanut Kanittakul, Sarawan Kaewmongkol, Gunn Kaewmongkol

**Affiliations:** 1 Kasetsart University International College (KUIC), Kasetsart University, Bangkok 10900, Thailand; peeravit.sum@ku.th; 2 Department of Veterinary Technology, Faculty of Veterinary Technology, Kasetsart University, Bangkok 10900, Thailand; kitjawan.khum@ku.th (K.K.); cvtswt@ku.ac.th (S.K.); 3 Internal Medicine Unit, Kasetsart University Veterinary Teaching Hospital, Faculty of Veterinary Medicine, Kasetsart University, Bangkok 10900, Thailand; krittanut.ka@ku.th; 4 Department of Companion Animals Clinical Sciences, Faculty of Veterinary Medicine, Kasetsart University, Bangkok 10900, Thailand

**Keywords:** isothermal amplification, canine monocytic ehrlichiosis, *Ehrlichia canis*

## Abstract

Canine monocytic ehrlichiosis (CME), caused by *Ehrlichia canis*, requires rapid, field-deployable diagnostics in endemic regions. This study developed and evaluated a recombinase polymerase amplification (RPA) assay for rapid isothermal detection of *E. canis* DNA using a dual-format gel-based approach. The diagnostic agreement of the RPA assay was evaluated by comparison with TaqMan qPCR and droplet digital PCR (ddPCR) using 41 well-characterized canine blood samples. The RPA assay yielded detectable amplification following an optimized incubation time of 30 min at 39 °C, combining agarose gel electrophoresis for visualization with amplicon recovery for sequencing. RPA detected *E. canis* in 25/41 samples (60.9%), identical to qPCR (60.9%) but lower than ddPCR (82.9%). Diagnostic evaluation showed moderate agreement between RPA and ddPCR (κ = 0.487, 95% CI: 0.233–0.741) and perfect agreement between RPA and qPCR (κ = 1.00). RPA demonstrated the same detection rate as qPCR in acute CME cases while using a low-cost heating device instead of specialized thermocyclers and identified positive cases without false-positive results. Sequencing of RPA amplicons confirmed 100% identity with the *E. canis dsb* gene reference sequence (AF403710.1). This RPA-based framework offers a flexible, dual-format approach that balances speed, accessibility, and diagnostic reliability, making it highly suitable for CME management in resource-limited settings.

## Simple Summary

Canine monocytic ehrlichiosis (CME) is an important tick-borne disease that requires rapid and accurate diagnosis for effective clinical management. Current molecular diagnostic methods are highly sensitive but often require expensive instruments and skilled personnel, limiting their use in many veterinary clinics and resource-limited settings. To address this challenge, we developed a recombinase polymerase amplification (RPA) assay that amplifies *Ehrlichia canis* DNA at a constant temperature of 39 °C using only a simple, inexpensive heating device and provides results within 30 min. The assay showed high agreement with established molecular methods when evaluated using a well-characterized clinical reference panel and did not generate false-positive results in the tested samples. In addition, gel-based detection allows recovery of amplification products for DNA sequencing, providing optional molecular confirmation. This study demonstrates a practical diagnostic workflow that combines rapid isothermal amplification with confirmatory sequencing, offering an accessible molecular diagnostic approach for CME in endemic and resource-limited regions.

## 1. Introduction

Globally, dogs face a major clinical threat from canine monocytic ehrlichiosis (CME), a tick-borne disease that is particularly severe in tropical regions, including Thailand and Southeast Asia. The causative agent of CME is *Ehrlichia canis*, a Gram-negative, intracellular bacterium belonging to the family *Anaplasmataceae* [1]. Because CME is associated with high rates of morbidity and mortality, it remains a primary concern for veterinary health in these regions [2,3,4]. A rapid, sensitive, specific, and accurate diagnostic method for CME is crucial for disease management and prevention of transmission.

The diagnosis of *E. canis* infection poses significant challenges because CME is associated with various clinical signs, including fever, anemia, and thrombocytopenia, which are similar to those seen in other blood pathogen infections [5,6]. Microscopic examination of blood smears for *E. canis* morulae remains a practical diagnostic approach in many local veterinary hospitals with resource-limited facilities, particularly in developing countries. However, this method has several limitations, including low diagnostic sensitivity, especially during the subclinical phase of infection [1,7,8]. In addition, this technique requires skilled examiners to differentiate *E. canis* infections from other cytoplasmic inclusions [9,10]. Serological diagnostic methods, such as the indirect immunofluorescence antibody (IFA) test or enzyme-linked immunosorbent assay (ELISA), are crucial in cases with clinical findings consistent with CME [11]. Nonetheless, these techniques cannot distinguish between species because of cross-reactivity among species-specific antibodies [12].

Molecular diagnostic techniques for CME, including conventional polymerase chain reaction (PCR) and real-time PCR (qPCR), provide high specificity and sensitivity for the detection of *E. canis* DNA [13,14]. Conventional PCR (cPCR) has been widely used for species identification by targeting the 16S rRNA gene and other conserved genetic loci [13,15,16,17,18]. Several advanced PCR-based assays have further improved the sensitivity of *E. canis* detection in laboratory settings [19,20]. However, these techniques are less suitable for point-of-care applications because they require specialized equipment, trained personnel, and relatively long turnaround times.

More recently, droplet digital PCR (ddPCR) has emerged as an advanced molecular technique that enables absolute DNA quantification and provides greater sensitivity than cPCR, with an approximately 78-fold lower limit of detection [8]. In addition, ddPCR reduces the impact of PCR inhibitors, allowing highly precise detection in samples with low bacterial loads [21]. Using the same well-characterized clinical reference panel employed in the present study, cPCR, qPCR, and ddPCR detected *E. canis* DNA in 53.7%, 61.0%, and 80.5% of blood samples, respectively, highlighting the superior sensitivity of ddPCR for CME [21]. However, its routine clinical application remains limited by the requirement for specialized instrumentation and experienced operators. In addition, ddPCR does not readily permit recovery of PCR amplicons for DNA sequencing confirmation, limiting its utility in sequence verification [22].

Several isothermal nucleic acid amplification methods have recently been developed, including loop-mediated isothermal amplification (LAMP), rolling circle amplification (RCA), strand displacement amplification (SDA), helicase-dependent amplification (HDA), and recombinase polymerase amplification (RPA). Among these methods, RPA has attracted considerable attention because of its high sensitivity, rapid amplification, operational simplicity, and minimal equipment requirements for the detection of zoonotic pathogens [23,24,25]. RPA operates at a constant temperature of 37–42 °C and typically generates detectable amplification products within 5–30 min through recombinase-mediated primer targeting and strand-displacement DNA synthesis [26].

In this study, we developed and evaluated a rapid method specific to *E. canis* using recombinase polymerase amplification targeting the disulfide bond formation protein (*dsb*) gene, and compared its diagnostic agreement with qPCR and ddPCR. In addition, RPA detection was performed using rapid visualization with agarose gel electrophoresis for amplicon recovery, followed by Sanger sequencing.

## 2. Materials and Methods

### 2.1. Ethical Considerations

The procedures for blood collection and the research methodology were approved by the Kasetsart University Institutional Animal Care Committee (ACKU67-VET-041) and the Animal Ethics Committee of the Faculty of Veterinary Medicine, Kasetsart University, Bangkok, Thailand. Written informed consent was obtained from all dog owners before enrollment. Blood samples were collected by licensed veterinarians as part of routine clinical diagnostic procedures, and only the remaining EDTA whole-blood samples were used for this study.

### 2.2. Animal Inclusion, Blood Sample Collection and DNA Extraction

Archived EDTA whole-blood samples from 41 client-owned dogs that had been prospectively enrolled at the Kasetsart University Veterinary Teaching Hospital (KUVTH), Bangkok, Thailand, between May and October 2024 were used in this study. All dogs presented with clinical signs consistent with canine monocytic ehrlichiosis (CME), for which CME was considered the primary clinical diagnosis, and were seropositive for *E. canis* using the IDEXX SNAP 4Dx Plus test (IDEXX Laboratories, Inc., Westbrook, ME, USA).

Following routine diagnostic testing, the remaining EDTA whole-blood samples were stored at −80 °C for future molecular investigations. For samples in which genomic DNA extracts generated during previous molecular studies [21] were still available, the archived DNA extracts were used for the present RPA analysis. For samples in which the previously extracted DNA had been exhausted, genomic DNA was newly extracted from the corresponding archived EDTA whole-blood samples using the E.Z.N.A. Tissue DNA Kit (Omega Bio-Tek, Norcross, GA, USA), according to the manufacturer’s instructions. All DNA samples used in the present study were subsequently stored at −20 °C until RPA analysis.

### 2.3. Molecular Detection

#### 2.3.1. RPA for *E. canis* Detection

RPA primers for amplifying the *dsb* gene of *E. canis* were selected from a previous study [14,19,21]. The RPA primers targeting the *dsb* gene were adopted from our previous studies, which were successfully validated for conventional PCR, qPCR, and ddPCR detection of *Ehrlichia canis*. Multiple sequence alignments confirmed that the primer-binding sites are located within a highly conserved region of the *dsb* housekeeping gene of local *E. canis* genotypes. The RPA reactions were performed for the DNA amplification of *E. canis* in a final volume of 50 µL using the TwistAmp^®^ Basic Kit (TwistDx, Maidenhead, UK). Each reaction contained 1× Rehydration Buffer and 0.48 µM of each forward and reverse primer (Table 1). The reaction mixture was prepared by combining the rehydration buffer, primers, and 5 µL of template DNA, adjusting the volume to 47.5 µL using nuclease-free water, before the mixture was added to the lyophilized enzyme pellet. The amplification was initiated by adding 2.5 µL of 280 mM magnesium acetate. The tubes were incubated at 39 °C for 30 min. Amplification time was optimized by testing incubation periods ranging from 10 to 30 min at 39 °C, with 30 min selected for all subsequent experiments because it consistently yielded the strongest amplification signal. The resulting RPA products obtained were analyzed by agarose gel electrophoresis. Each RPA assay included a positive control containing *E. canis* DNA and a no-template control (NTC), which were processed alongside the clinical samples. Representative RPA amplicons were randomly selected, purified, and submitted to First BASE Laboratories Sdn. Bhd. (Seri Kembangan, Selangor, Malaysia) for Sanger sequencing. Sequencing was performed using BigDye^®^ Terminator v3.1 chemistry on an ABI PRISM 3730xl Genetic Analyzer (Applied Biosystems, Foster City, CA, USA). Chromatograms were inspected for sequence quality, and the resulting consensus sequences were compared with reference sequences in the GenBank database using BLASTn to confirm the identity of *E. canis*. The obtained *dsb* gene sequences were subsequently submitted to the GenBank database under accession numbers PZ518726–PZ518729.

#### 2.3.2. TaqMan Real-Time PCR for *E. canis* Detection

Blood samples from dogs were analyzed by qPCR assay as previously described by Sumpavong et al. [21]. The assay specifically targeted the *dsb* gene. Each 20 µL reaction mixture comprised HOT FIREPol Probe qPCR Mix Plus (Solis BioDyne, Tartu, Estonia), 1 µM of each primer, and the TaqMan probe (Table 1). The CFX96 Real-Time PCR System (Bio-Rad Laboratories, Hercules, CA, USA) was used for qPCR. Thermocycling conditions consisted of initial denaturation at 95 °C for 15 min, followed by 45 cycles at 95 °C for 15 s and 60 °C for 1 min.

#### 2.3.3. Droplet Digital PCR for *E. canis* Detection

Blood samples from dogs were analyzed using a ddPCR assay as previously described by Sumpavong et al. [21]. The QX200™ Droplet Digital PCR system (Bio-Rad Laboratories, Hercules, CA, USA) was used in this research in accordance with the manufacturer’s guidelines. Each ddPCR reaction mixture (20 µL) included 1X ddPCR Supermix for Probes (Bio-Rad), 900 nM of each primer, 250 nM of the probe (Table 1), and 2 µL of the DNA sample. The PCR mixture (20 µL), along with 70 µL of droplet generation oil for probes, was introduced into the sample and oil wells of an 8-channel DG8 cartridge positioned in its appropriate holder.

Droplets were generated using the QX200TM Droplet Generator (Bio-Rad) following the manufacturer’s guidelines. The droplets were transferred into a ddPCR^TM^ 96-well plate (Bio-Rad) by aspirating 40 µL from the DG8 cartridge. The plate was sealed with pierceable foil using a PX1TM PCR Plate Sealer (Bio-Rad) at 180 °C for 5 s and amplified on a C1000 Touch^TM^ Thermal Cycler (Bio-Rad). The optimized PCR conditions involved an initial denaturation at 95 °C for 10 min, followed by 45 cycles of 94 °C for 30 s and annealing/extension at 60 °C for 1 min and 30 s (temperature ramping at 2 °C/s), with a final incubation at 98 °C for 10 min and storage at 4 °C. After amplification, the plate was moved to a QX200^TM^ Droplet Reader (Bio-Rad). Each droplet from the samples was analyzed separately, and fluorescence signals were assessed using QX Manager Software 2.2 (Bio-Rad).

### 2.4. Detection Limit of the Molecular Assays

A recombinant plasmid harboring the *dsb* gene of *E. canis* was prepared as described previously [14]. The purified plasmid was quantified, and the nominal copy concentration was calculated from the DNA concentration and total plasmid length. The plasmid working stock was then subjected to consecutive ten-fold serial dilutions (10^1^–10^−8^ relative to the working stock), which were used to compare the analytical detection endpoints of RPA, qPCR, and ddPCR.

The diagnostic agreement of the RPA assay was compared with TaqMan probe-based real-time PCR (qPCR) and droplet digital PCR (ddPCR) using 41 canine blood samples constituting a well-characterized clinical reference panel, previously confirmed and quantified by ddPCR, qPCR, and a commercial rapid lateral flow immunoassay [21]. In this comparative evaluation, ddPCR served as the highly sensitive comparator molecular assay for evaluating the relative diagnostic performance of RPA and qPCR. Accordingly, the relative sensitivity, relative specificity, positive predictive value, and negative predictive value were calculated using ddPCR as the comparator assay.

### 2.5. Statistical Analysis

Statistical analyses were performed using NCSS 2024 (version 24.0.2). Agreement between each pair of molecular assays (RPA vs. qPCR, RPA vs. ddPCR, and qPCR vs. ddPCR) was quantified using Cohen’s kappa statistic. Additionally, the relative diagnostic performance of the RPA assay was evaluated by calculating relative sensitivity, relative specificity, positive and negative predictive values, and likelihood ratios. In these analyses, qPCR and ddPCR were each evaluated separately as comparator molecular assays. Because no universally accepted molecular gold standard currently exists for the diagnosis of canine monocytic ehrlichiosis, these parameters should be interpreted as measures of agreement with the comparator assay rather than estimates of absolute diagnostic accuracy.

## 3. Results

### 3.1. Detection Limit and Assay Accuracy

The detection limit of the developed RPA assay was evaluated using 10-fold serial dilutions of a DNA plasmid harboring the *dsb* gene of *E. canis*, ranging from 10^1^ to 10^−8^. The RPA assay produced detectable amplification up to the 10^−7^ dilution (see Supplementary Materials Figure S1) following incubation at 39 °C for 30 min. Therefore, RPA and qPCR showed identical endpoint detection, and RPA detected one additional ten-fold dilution compared with conventional PCR (10^−6^ dilution), as demonstrated in a previous study (see Supplementary Materials Figure S2, Table S1) [14].

To evaluate assay accuracy, the RPA was challenged with genomic DNA from dog blood. Sequence analysis of the positive amplicons revealed a high degree of genetic similarity, 100% identity with the *E. canis* reference genotype (GenBank accession number: AF403710.1). These findings confirm the identity of the amplified target *dsb* gene under isothermal conditions.

### 3.2. Diagnostic Agreement

**Figure 1:**
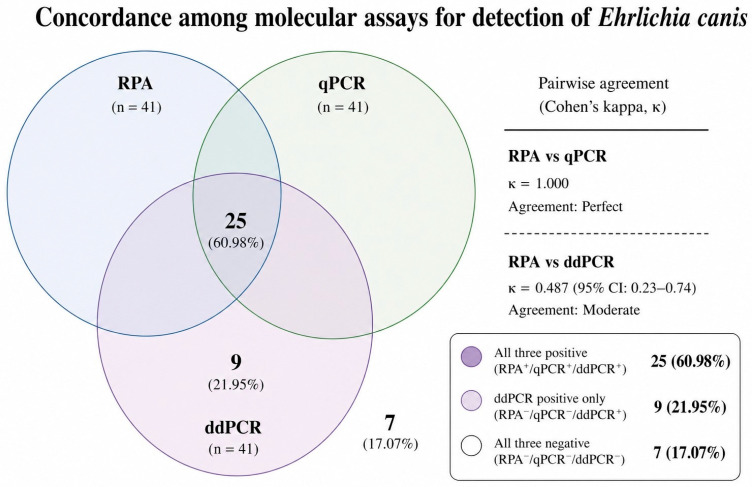
Venn diagram illustrating the overlap of positive detections among recombinase polymerase amplification (RPA), quantitative PCR (qPCR), and droplet digital PCR (ddPCR) for *Ehrlichia canis* detection. Numbers indicate the number of samples in each category, and percentages are based on the total study population (*N* = 41).

Of the 41 clinical blood samples, the RPA assay identified 25 positive cases (Table 2), demonstrating a high degree of concordance with both qPCR and ddPCR results. Statistical analysis revealed a Cohen’s kappa coefficient of 1.000 (Table 3), indicating a perfect agreement between RPA and qPCR. However, ddPCR detected 34 positive cases, suggesting that ddPCR was able to identify samples with low bacterial loads that were not detected by RPA or qPCR. A Venn diagram was constructed to visualize the concordance among the three molecular assays (Figure 1). All 25 RPA-positive samples were also positive by qPCR, whereas ddPCR detected an additional nine positive samples that were negative by both RPA and qPCR. Seven samples were negative by all three methods (Figure 1).

### 3.3. Statistical Results

#### 3.3.1. Concordance Among RPA, qPCR and ddPCR

Statistical analyses of data from RPA, qPCR, and ddPCR were performed using NCSS 2024 (version 24.0.2). The concordance between methods was assessed using Cohen’s kappa (κ) statistic (Table 3). The RPA assay demonstrated perfect agreement with qPCR (κ = 1.000), whereas it exhibited moderate agreement with ddPCR (κ = 0.487; 95% CI: 0.233–0.741). Similarly, the comparison between qPCR and ddPCR revealed moderate agreement (κ = 0.487; 95% CI: 0.233–0.741).

#### 3.3.2. Comparative Diagnostic Sensitivity and Specificity

The comparative diagnostic agreement of the RPA assay against qPCR and ddPCR is shown in Table 4. Compared with qPCR, RPA achieved 100% sensitivity (95% CI: 86.28–100%) and 100% specificity (95% CI: 79.41–100%). Compared with ddPCR, RPA sensitivity decreased to 73.5% (95% CI: 55.64–87.12%) while maintaining 100% specificity (95% CI: 59.04–100%). The positive predictive value (PPV) for RPA across both comparisons was 100% (95% CI: 86.28–100%), whereas the negative predictive value (NPV) was higher against qPCR (100%, 95% CI: 79.41–100%) than against ddPCR (43.8%, 95% CI: 19.75–70.12%). Regarding likelihood ratios, the RPA assay yielded positive (PLR) and negative (NLR) likelihood ratios of 32.00 and 0.02, respectively, against qPCR. In comparison with ddPCR, these values were 10.29 and 0.26, respectively.

## 4. Discussion

The development of the Recombinase Polymerase Amplification (RPA) assay in this study addresses a critical gap in the molecular diagnosis of *Ehrlichia canis*, particularly for point-of-care applications. The primary strength of RPA lies in its sophisticated enzymatic machinery, which utilizes a recombinase–primer complex to actively scan double-stranded DNA and facilitate strand displacement at a constant temperature. This process, stabilized by single-stranded binding (SSB) proteins and driven by a strand-displacing DNA polymerase, effectively mimics natural cellular replication and eliminates the need for rapid thermal cycling required by traditional PCR [26].

The robustness of the RPA assay developed in this study is supported by its consistent performance across different molecular assays. The use of a ddPCR-characterized sample panel is a strength of this study because it allowed the RPA assay to be evaluated against samples with known infection status and quantified bacterial load, rather than against unclassified clinical specimens. The higher detection rate of ddPCR may have clinical significance because this study population has previously been shown to exhibit an association between higher *E. canis* DNA loads, more severe anemia, and persistent DNAemia following doxycycline treatment [21]. This approach provided a rigorous benchmark for determining the clinical utility and limitations of RPA, particularly in low-load infections. The key finding was the perfect concordance (κ = 1) between RPA and qPCR. All 41 clinical samples produced identical qualitative results, with 100% relative sensitivity and specificity compared with qPCR. The moderate agreement (κ = 0.487) observed between RPA and ddPCR highlights a distinct sensitivity gap, as evidenced by nine discordant samples that were ddPCR-positive but RPA-negative. This discrepancy is likely attributable to the superior analytical sensitivity of ddPCR, which partitions the reaction into thousands of individual nanoliter droplets. This partitioning effectively reduces competition from background genomic DNA and enables absolute quantification of extremely low-copy targets that may fall below the detection threshold of isothermal chemistries [27]. Specifically, our findings indicate that while RPA serves as an excellent rapid screening tool, its lower sensitivity in low-load samples necessitates that negative results in highly symptomatic dogs be followed by a confirmatory quantitative assay (such as ddPCR).

The relatively low negative predictive value (43.8%) observed when ddPCR was used as the comparator assay indicates that an RPA-negative result cannot reliably exclude the presence of *E. canis* DNA, particularly in samples with low bacterial loads. This finding likely reflects the higher sensitivity of ddPCR and suggests that negative RPA results should be interpreted cautiously when molecular confirmation is clinically warranted. Although no false-positive RPA results were observed, resulting in a high positive likelihood ratio, the confidence interval was wide because of the limited number of ddPCR-negative samples and the absence of false-positive observations. Therefore, these estimates should be interpreted cautiously.

The RPA assay is also highly flexible, enabling the creation of a diagnostic framework tailored to diverse clinical and field-based needs. The combination of RPA and Lateral Flow Assays provides a visual positive/negative result suitable for rapid, on-site surveillance and initial screening in the field [28]. By contrast, when definitive molecular confirmation or epidemiological tracking is necessary, the utilization of RPA followed by agarose gel electrophoresis is essential. This method allows specific amplicons to be visualized directly, and Sanger sequencing can then confirm them. The 100% sequence identity of the *E. canis* reference sequence (AF403710.1) observed in this study confirms that the RPA-based workflow is not only fast but also maintains high genetic fidelity. This dual-format RPA approach is a robust and flexible way to manage canine monocytic ehrlichiosis by bridging the gap between field-based utility and laboratory-grade accuracy [29]. Recombinase polymerase amplification (RPA) is known to tolerate primer–template mismatches and lacks intrinsic proofreading activity, which may increase the likelihood of sequence variation in amplified products. Therefore, nucleotide discrepancies observed in RPA-derived amplicons, such as unexpected stop codons, should be interpreted with caution and ideally confirmed using high-fidelity PCR-based methods [30].

Analytical specificity was not evaluated against an empirical exclusivity panel of closely related pathogens, which is a limitation of this study. Although primer specificity was supported by previous validation [19], in silico analysis, and amplicon sequencing, future studies should include cross-reactivity testing to confirm assay specificity.

Difficulty in developing new molecular techniques is common in most infectious diseases because these diseases lack a molecular gold standard. The accuracy of the tests could only be assessed by comparing them with previously reported tools that serve as molecular comparators rather than gold-standard molecular tools [31]. The complete concordance between RPA and qPCR indicates that the newly developed assay performs comparably to the current routine molecular diagnostic approach. Although ddPCR identified additional low-bacterial-load infections due to its superior analytical sensitivity, it was used in this study as a highly sensitive comparator assay rather than as an absolute molecular gold standard.

## 5. Conclusions

The present study demonstrates that the *dsb*-targeted RPA assay provides rapid molecular detection of *Ehrlichia canis* with a detection rate and diagnostic agreement comparable to qPCR while substantially reducing assay time and equipment requirements. Although ddPCR remains the most analytically sensitive method for detecting very low bacterial loads, the RPA assay offers a practical qualitative alternative for routine molecular detection. Furthermore, the gel-based RPA approach enables recovery of amplification products for sequence confirmation, providing an additional advantage for molecular confirmation. Consequently, these findings support the potential application of RPA as a rapid and accessible molecular diagnostic tool for *E. canis*, particularly in laboratories with limited molecular infrastructure.

## Figures and Tables

**Table 1:** Nucleotide sequences of the primers and TaqMan probe targeting the *dsb* gene of *E. canis*.

Oligo	Sequence 5′–3′	Reference
*dsb*-F	TTGCAAAATGATGTCTGAAGATATGAAACA	[19]
*dsb*-R	GCTGCTCCACCAATAAATGTATCYCCTA	
DNA probe	FAM-5′-AGCTAGTGCTGCTTGGGCAACTTTGAG TGAA-BHQ1-3′	

**Table 2:** Comparative diagnostic agreement of ddPCR, qPCR, and RPA assays for the detection of *E. canis* in clinical samples.

Sample	RPA		qPCR		ddPCR	
	Positive	Negative	Positive	Negative	Positive	Negative
41 Samples	25 (60.97%)	16	25 (60.97%)	16	34 (82.92%)	7

**Table 3:** (**A**) Pairwise concordance of RPA and qPCR with ddPCR. (**B**) Concordance between RPA and qPCR.

**(A) Pairwise concordance of RPA and qPCR with ddPCR**					
		**ddPCR**		**Kappa Value** **(95% CI)**	**Degree of agreement**
		Positive	Negative		
**RPA**	Positive	25	0	0.487 (0.23–0.74)	Moderate
	Negative	9	7		
**qPCR**	Positive	25	0	0.487 (0.23–0.74)	Moderate
	Negative	9	7		
**(B) Concordance between RPA and qPCR**					
		**qPCR**		**Kappa Value** **(95% CI)**	**Degree of agreement**
		Positive	Negative		
**RPA**	Positive	25	0	1	Perfect
	Negative	0	16		

**Table 4:** Relative diagnostic performance of the RPA assay using qPCR and ddPCR as comparator molecular assays.

Diagnostic Performance	qPCR (95% CI)	ddPCR (95% CI)
Relative Sensitivity	100% (86.28–100)	73.53% (55.64–87.12)
Relative Specificity	100% (79.41–100)	100% (59.04–100)
Positive predictive value	100% (86.28–100)	100% (86.28–100)
Negative predictive value	100% (79.41–100)	43.75% (19.75–70.12)
Positive likelihood ratio	32.00 (2.09–∞*)	10.29 (0.71–∞*)
Negative likelihood ratio	0.02 (0.00–0.31)	0.26 (0.15–0.46)

∞*, infinity. The upper limit of the 95% CI for the positive likelihood ratio could not be determined due to a specificity of 100%.

## Data Availability

The original contributions presented in this study are included in the article/Supplementary Materials. Further inquiries can be directed to the corresponding author.

## Supplementary Materials

The following supporting information can be downloaded at https://www.mdpi.com/article/10.3390/ani16152443/s1, Figure S1: Detection limit of the RPA assay for *E. canis* detection. Ten-fold serial dilutions of DNA plasmid harboring *dsb* gene of *E. canis* were used as templates.; Figure S2: Comparison of limitations of three molecular assays using 10-fold serial dilutions; Table S1: Endpoint detection of RPA, qPCR, and conventional PCR using ten-fold serial dilutions of the pGEMT-dsb plasmid. References [14,21] are cited in the Supplementary Materials.
