# Supplementary material for: Rapid Gel-Based Recombinase Polymerase Amplification Assay for Detection and Sequence Confirmation of Ehrlichia canis
Source: Animals (Basel). 2026 Aug 6;16(15):2443. doi: 10.3390/ani16152443 (PMC13465801; doi:10.3390/ani16152443)
Supplement: Supplementary file 1 [file animals-16-02443-s001.zip › animals-4435884-supplementary.pdf]

Supplementary Materials S1

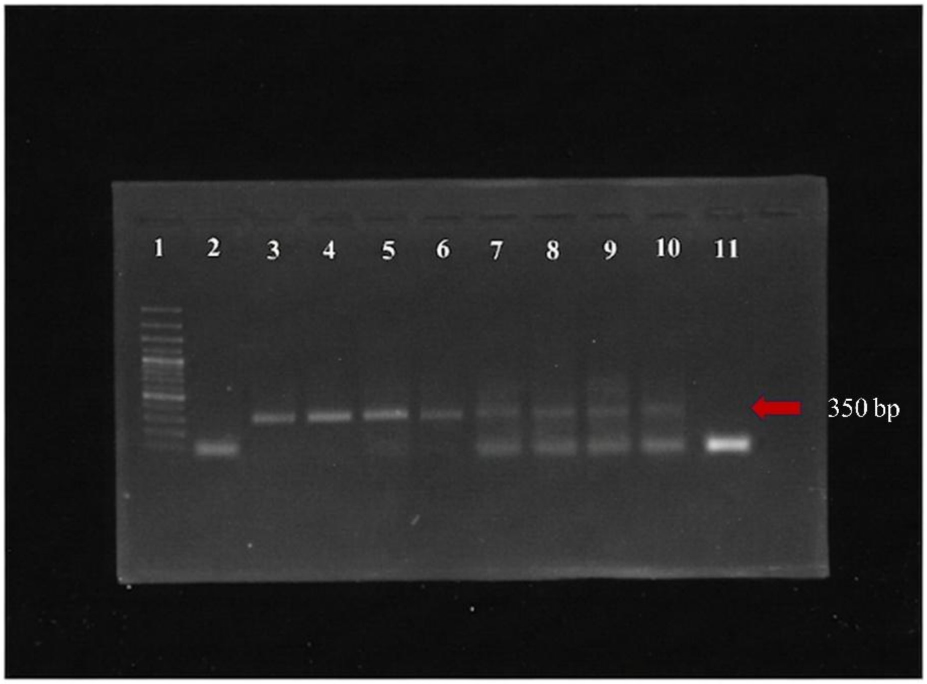

Figure S1. Detection limit of the RPA assay for *E. canis* detection. Ten-fold serial dilutions of DNA plasmid harboring *dsb* gene of *E. canis* were used as templates. Lane 1, DNA marker; Lane 2, non-template control, Lanes 3–11, serial ten-fold dilutions (10^0 to 10^{-8}) of the plasmid working stock.

Supplementary Materials S2

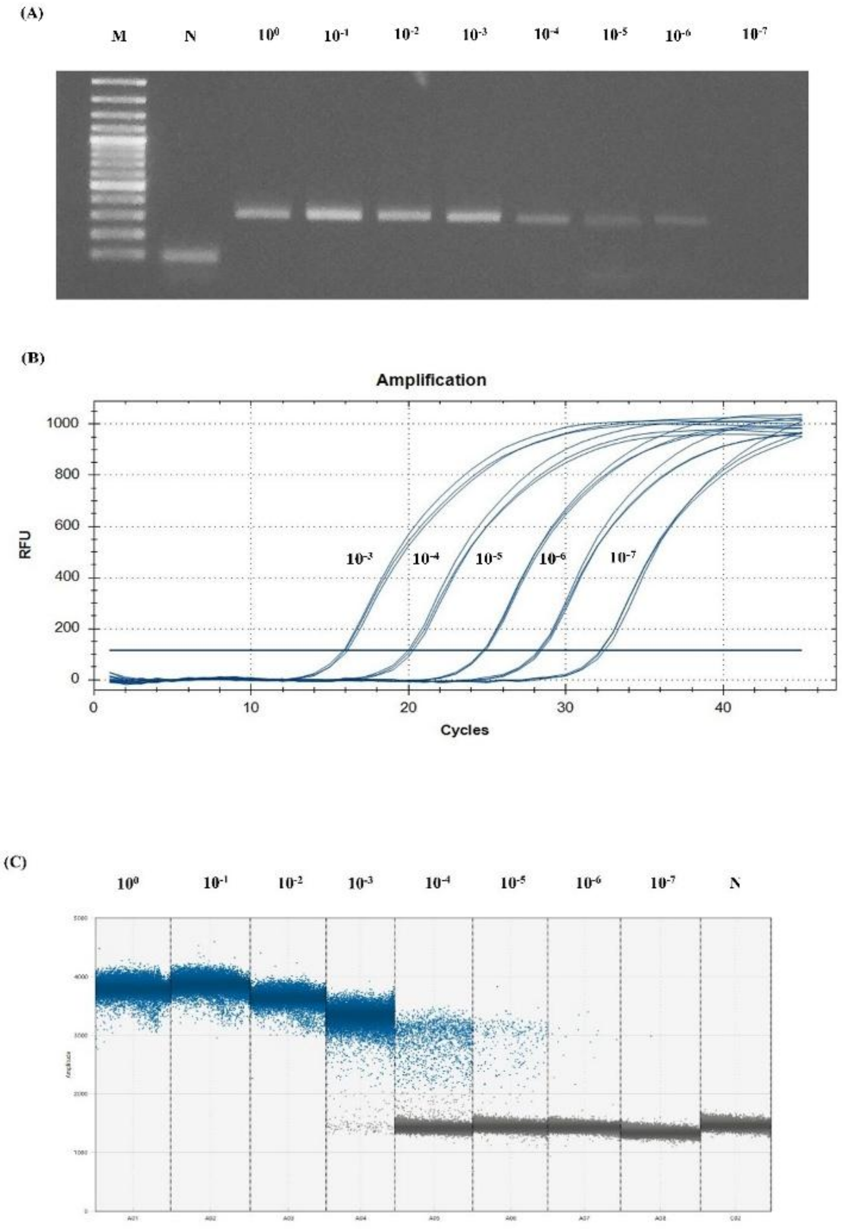

Figure S2. Comparison of limitations of three molecular assays using 10-fold serial dilutions (A) Agarose gel electrophoresis of cPCR Lane M: Marker (Gene Ruler 100 bp plus) Lane N: Negative control and Lane 3-10: 10-fold pGEM-T-*dsb* dilution (Sumpavong et al., 2022), (B) The standard curve generated by qPCR (Sumpavong et al., 2022), (C) ddPCR absolute quantification of ddPCR (Sumpavong et al., 2026).

Supplementary Materials S3

Table S1. Endpoint detection of RPA, qPCR, and conventional PCR using ten-fold serial dilutions of the pGEMT-*dsb* plasmid.

**Molecular assay**	**Lowest detectable plasmid dilution**	**Source**
ddPCR	10^{-7}	Sumpavong et al. (2026)
qPCR	10^{-7}	Sumpavong et al. (2022)
cPCR	10^{-6}	Sumpavong et al. (2022)
RPA	10^{-7}	Present study
